# Identification of W13 in the American Miniature Horse and Shetland Pony Populations

**DOI:** 10.3390/genes12121985

**Published:** 2021-12-14

**Authors:** Elizabeth Esdaile, Angelica Kallenberg, Felipe Avila, Rebecca R. Bellone

**Affiliations:** 1 Veterinary Genetics Laboratory, School of Veterinary Medicine, University of California–Davis, Davis, CA 95616, USA; esesdaile@ucdavis.edu (E.E.); akallenberg@ucdavis.edu (A.K.); ffavila@ucdavis.edu (F.A.); 2 Department of Population Health and Reproduction, School of Veterinary Medicine, University of California–Davis, Davis, CA 95616, USA

**Keywords:** *KIT*, dominant white, white spotting, coat color, pigmentation, horse

## Abstract

Coat color is a trait of economic significance in horses. Variants in seven genes have been documented to cause white patterning in horses. Of the 34 variants that have been identified in KIT proto-oncogene, receptor tyrosine kinase (*KIT*), 27 have only been reported in a single individual or family and thus not all are routinely offered for genetic testing. Therefore, to enable proper use of marker-assisted selection, determining breed specificity for these alleles is warranted. Screening 19 unregistered all-white Shetland ponies for 16 white patterning markers identified 14 individuals whose phenotype could not be explained by testing results. In evaluating other known dominant white variants, 14 horses were heterozygous for W13. W13 was previously only reported in two quarter horses and a family of Australian miniature horses. Genotyping known white spotting variants in 30 owner-reported white animals (25 Miniature Horses and five Shetland ponies) identified two additional *W13/N* American Miniature Horses. The estimated allele frequency of W13 in the American Miniature Horse was 0.0063 (79 *N/N*, 1 *W13/N*) and the allele was not detected in a random sample (*n* = 59) of Shetland ponies. No homozygous W13 individuals were identified and *W13/N* ponies had a similar all-white coat with pink skin phenotype, regardless of the other white spotting variants present, demonstrating that W13 results in a Mendelian inherited dominant white phenotype and homozygosity is likely lethal. These findings document the presence of W13 in the American Miniature Horse and Shetland pony populations at a low frequency and illustrate the importance of testing for this variant in additional breeds.

## 1. Introduction

Coat color is a trait of economic significance and was one of the first traits to be investigated in horses [1,2,3,4]. Variants that impact base coat color have been identified in agouti signaling protein (*ASIP*) and melanocortin 1 receptor (*MC1R*) [5,6,7]. Six variants in five genes have been shown to dilute pigment, and these are known as cream (Cr), pearl (Prl), champagne (Ch), dun (D), mushroom (Mu), and silver (Z) [8,9,10,11,12,13]. Variants in seven genes (*KIT*, *EDNRB*, *TRPM1*, *MITF*, *PAX3*, *RFWD3*, *STX17*) have been documented to cause white patterning in domestic horses [14,15,16,17,18,19,20,21,22,23,24,25,26,27,28,29,30,31,32,33,34,35]. The majority of the white patterning variants described are within or are thought to regulate the KIT proto-oncogene, receptor tyrosine kinase (*KIT*) [14,15,16,17,18,19,20,21,22,23,24,25,26,27,28]. These variants explain several different but overlapping phenotypes including sabino-1 (SB1), tobiano (TO), and dominant white (W1–W17a, W17b–W28, W30–32) [14,15,16,17,18,19,20,21,22,23,24,25,26,27,28]. The sabino phenotype is characterized by white patterning on the face and legs and white patches and/or white hairs dispersed on the belly that can extend upward on the barrel [14]. Most commonly, the sabino phenotype results from heterozygous expression of an intronic SNP in *KIT* that impacts splicing, named “Sabino-1” (SB1). Horses homozygous for this variant are nearly all white. Tobiano is caused by a large inversion thought to disrupt KIT expression and has been described as a nonsymmetrical white patterning on the body that usually crosses the top line with white lower legs and hooves [15]. In contrast to SB1, homozygous tobiano horses do not display an all-white phenotype. Dominant white variants are thought to produce a range of phenotypes from a minimal sabino-like phenotype to a horse that is completely white. In the heterozygous state, W5, W10, W12, W19, W21, W22, W26, W31, and W32 have been reported to cause a sabino-like phenotype [17,18,20,21,22,25,28]. W20 is thought to have a subtler impact on white patterning but, in the presence of other known white-causing alleles, modifies the extent of white patterning [20]. In the strictest sense, those variants termed dominant white were originally reserved for *KIT* alleles thought to be homozygous lethal [16], but there are known exceptions including W15, W20, and W32 [19,20,28].

In addition to mutations impacting the *KIT* locus, variants in six other genes have been shown to cause an all-white or nearly all-white pattern. Lethal white overo is a white spotting phenotype that is the result of a two-nucleotide substitution in the endothelin B receptor gene (*EDNRB*, designated as O for overo) [29]. In heterozygotes, overo manifests as a jagged white spotting phenotype across the body, neck, and legs of the horse and large white markings on the face, sometimes resulting in deafness. Homozygotes have an all-white coat and typically die shortly after birth due to ileocolonic aganglionosis [29]. In Appaloosas and related breeds with leopard complex spotting, homozygosity for a large insertion within the calcium ion channel gene transient receptor potential cation channel subfamily M member 1 (*TRPM1*, commonly referred to as LP) inherited along with a SNP in the 3’ untranslated region of ring finger and WD repeat domain 3 *(RFWD3*, known as PATN1 for LP first pattern modifier) is thought to cause a nearly all-white phenotype, referred to as “few-spot”. Heterozygosity for LP results in a white spotting pattern with oval spots of pigment in the white patterned area [30,34]. Six alleles in two transcription factors that regulate melanogenesis, namely melanocyte inducing transcription factor (*MITF*) and paired box 3 (*PAX3*), contribute to the splash white (SW) phenotype characterized by extensive depigmentation of the head, legs, and some white spotting on the belly. Homozygosity for a 10 bp insertion in the melanocyte-specific promoter of *MITF*, known as SW1, produces a range in the extent of white patterning but in some cases has been shown to result in a nearly all-white coat phenotype [31]. Compound heterozygosity for the promoter mutation (SW1) and a frame shift mutation in this same gene (p.C280Sfs*20, SW3) is thought to cause an all-white phenotype [31]. Homozygosity for the SW1 variant in *MITF*, along with a copy of an identified missense mutation in *PAX3* (p.C70Y, SW2), also causes an all-white phenotype [31]. Finally, unlike the other white patterning phenotypes, gray is progressive with age and can eventually result in an all-white hair coat with pigmented skin [35]. Gray is caused by a dominant 4.6-kilobase duplication in an intron of the gene syntaxin 17 (*STX17*, denoted as G) [35].

Horse breeders selectively breeding for white patterning often use genetic testing for marker-assisted selection for the variants described above. Twenty-seven of the dominant white variants at the *KIT* locus have been reported in a single individual or family, thus genetic testing for most of the dominant white variants is not routinely performed. However, in cases where phenotypes are not explained by reported results, additional information on both known and novel variants can help inform breeding decisions. In this study, to assist in breeding decisions, we aimed to identify the cause of all-white coat phenotypes in American Miniature Horses and Shetland ponies not explained by routine genetic testing results.

## 2. Materials and Methods

Hair and blood samples as well as photographs for 19 all-white patterned unregistered Shetland ponies were provided by Tri-Circle-D Ranch at the Walt Disney World Resort^®^ (group denoted as “unregistered Shetland ponies”). Coat color and the presence or absence of skin pigmentation in the muzzle and orbital region were phenotyped with the images provided. Ponies were classified as either all white with pink skin, all white with dark skin, or all white with evidence of pigmented and unpigmented skin. Hair samples for 30 owner-reported white animals (5 Shetland ponies and 25 Miniature Horses) banked at the University of California, Davis Veterinary Genetics Laboratory were also utilized to investigate the cause of white patterning within the breeds under investigation.

DNA from blood, mane, or tail hair was extracted using the Qiagen Gentra Puregene Blood Kit (Germantown, MD, USA) as previously described [36] or with a crude hair lysis protocol as described by Locke et al. [37]. Genotyping for equine coat color loci routinely tested at the University of California, Davis Veterinary Genetic Laboratory was performed by the service section of the laboratory for base coat color, including *ASIP* (A or a) and *MC1R* (E, e^a^, e); variants that cause dilution of pigment, including champagne (Ch), cream (Cr), pearl (Prl), dun (D, nd1, nd2), mushroom (Mu), and silver (Z); and 16 white spotting alleles, including sabino-1 (SB1), tobiano (TO), dominant white 5, 10, 20, 22 (W5, W10, S20, W22), lethal white overo (O), leopard complex (LP), pattern-1 (PATN1), splashed whites 1–6 (SW1–SW6), and the presence, absence, or zygosity of gray (G) (https://vgl.ucdavis.edu/panel/full-coat-color-pattern-panel, https://vgl.ucdavis.edu/test/gray, and https://vgl.ucdavis.edu/test/mushroom, all accessed on 12 December 2021). Horses were also genotyped for additional dominant white alleles by one of three different methodologies. W1–W4, W6–W9, W11–W19, W21, W23–25 were genotyped using an Ion Torrent S5 genotyping by sequencing (GBS) assay (Thermo Fisher Scientific, Austin, TX, USA) that included an AgriSeq™ HTS Library Kit and a custom-designed amplicon panel. Genotypes for W26, W27, and W30 were determined using the Agena Bioscience iPLEX Gold reagents and MassARRAY assay (San Diego, CA, USA). Genotypes for W28 were identified using a fluorescently tagged allele-specific PCR assay and visualized on the ABI3730. Specific details of all dominant white variants are summarized in the Online Mendelian Inheritance in Animals database (https://omia.org/OMIA000209/9796/ accessed on 12 December 2021).

One hundred thirty-nine randomly selected samples (59 Shetland ponies and 80 American Miniature Horses) with unknown phenotypes banked at the University of California, Davis Veterinary Genetics Laboratory were also screened to estimate the allele frequency for the detected W13 allele within the breeds under investigation. The allele frequency of W13 was calculated using Microsoft Excel.

## 3. Results

**Figure 1:**
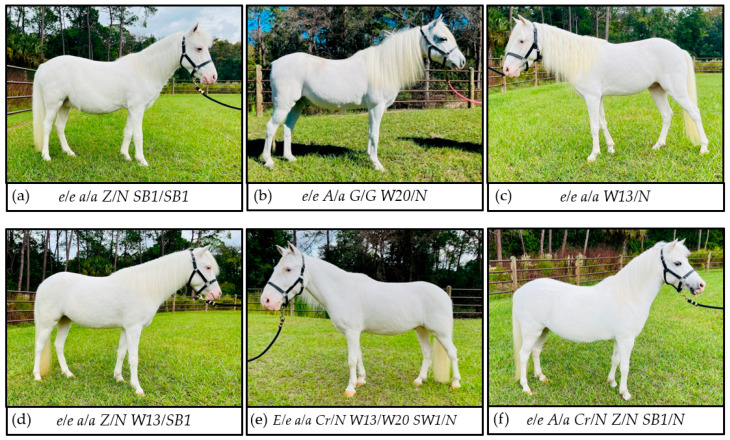
Unregistered Shetland ponies with an all-white coat phenotype and genetic causes. Genotypes and phenotypes are as follows: (**a**) Chestnut base coat (*e/e a/a Z/N*) with all-white coat phenotype and pink skin explained by homozygosity for *SB1/SB1*; (**b**) chestnut base coat (*e/e A/a*) with white coat and dark skin phenotype explained by gray (*G/G*) and *W20/N*; (**c**) chestnut base coat (*e/e a/a*) with all-white coat phenotype explained by *W13/N*; (**d**) chestnut base coat (*e/e a/a Z/N*) and compound heterozygote at the *KIT* locus *W13/SB1*; (**e**) black base coat (*E/e a/a Cr/N*) with *W13/W20* at the *KIT* locus and with the SW1 allele (*SW1/N*); (**f**) palomino base coat (*e/e A/a Cr/N Z/N*) with *SB1/N* that does not explain the all-white phenotype.

Of the 19 all-white patterned unregistered Shetland ponies, only the phenotypes of four were explained by genetic variants commonly tested by the UC Davis Veterinary Genetics Laboratory (2 *SB1/SB1*, 2 *G/G*, Table 1 and Table 2). Genotyping the 15 individuals with unexplained all-white phenotypes for 26 additional dominant white variants identified 14 ponies heterozygous for W13 (NC_009146.3:g.79544066C>G, ENSECAT00000014185.3:c.2807+5G>C) (Table 1 and Table 3). Seven of these individuals had no known relation to each other and seven were offspring of a single sire. All individuals with W13 had an all-white coat pattern and pink skin, five of these had no other known mutations, and nine had other white patterning mutations that by themselves do not cause an all-white phenotype (Figure 1, Table 3). Two horses with *LP/N* and *PATN1/N* genotypes, typically indicative of a large amount of white patterning with oval spots of pigment in the white patterned area, were also heterozygous for W13 (*W13/N*) and had an all-white coat phenotype. Therefore, it does not appear that W13 acts in an additive fashion with other white spotting patterns but rather as a dominant trait, creating an all-white phenotype that is epistatic to other genetic markers. One individual with an all-white hair coat and heterogeneous pigmented and unpigmented skin was heterozygous for SB1 but did not have any of the other white patterning alleles (Table 1, Figure 1). Heterozygosity for SB1 (*SB1/N*) alone does not explain the extensive depigmentation observed.

In evaluating the genotypes of 25 owner-reported white American Miniature Horses, 20 were explained by routine genetic testing results for white spotting variants including gray (Table 1 and Table 2). Five were homozygous for SB1 or were homozygous for this variant while also having additional white variants at other loci. Seven were determined to have at least one copy of the gray allele or gray combined with other white patterning alleles. Eight were explained by a combination of multiple white spotting pattern alleles (Table 2). Five of the American Miniature Horses could not be explained by routine genetic testing results; two of these were heterozygous for the W13 allele, and three horses could not be explained by any of the additional variants screened (Table 1 and Table 2).

All of the owner-reported white Shetland ponies (*n* = 5) could be explained by routine testing for gray (*n* = 3), Sabino-1 (*SB1/SB1*, *n* = 1), or a combination of white spotting variants (*n* = 1) (Table 2). None had the W13 allele that was detected in the unregistered Shetland ponies with an all-white coat phenotype.

Given the presence of W13 in the American Miniature Horse and the Shetland pony, we calculated the allele frequency by genotyping a random sample set for each breed. The allele frequency of W13 in Miniature Horses was estimated to be 0.0063 (79 *N/N*, 1 *W13/N,* Table 4) and W13 was not identified in randomly selected Shetland ponies (Table 4). Additionally, no individuals were homozygous for W13.

## 4. Discussion

In evaluating a total of 49 horses for causes of an all-white coat pattern, four horses’ phenotypes were unexplained. It is possible that these represent novel causes for dominant white in the Shetland and miniature pony breeds. Performing whole-genome sequencing on these horses with photographic records to evaluate *KIT* and other functional candidate genes could help to unravel the genetic mechanism/s in these cases.

Phenotypes of sixteen horses whose all-white coats were not explained by variants routinely tested at the UC Davis Veterinary Genetics Laboratory could be explained by heterozygosity for W13. One additional *W13/N* Miniature Horse was identified when screening a random sample set of miniature horses. While the estimated W13 allele frequency in the Shetland and Miniature populations was low, given selection for white patterning and the number of *W13/N* individuals on a single breeding farm (*n* = 14), if homozygosity was viable we likely would have detected a homozygous individual in this sample set. Therefore, these data provide further evidence of W13 homozygous embryonic lethality like that of W1–W14, W16, W17a, W17b, W18, W19, and W21–W31 [16,17,18,19,20,21,22,23,24,25,26,27,28]. Given that the Shetland ponies reported here with the W13 allele were unregistered and that we did not detect the W13 allele when screening 57 registered Shetland ponies, it is possible that the unregistered Shetland ponies in this study may have some American Miniature Horse introgression. Additional genotyping of all-white registered Shetland ponies is necessary to fully investigate W13 within the breed.

Previous studies only identified the W13 allele in six animals. The first report identified only two all-white, *W13/N*, individuals as quarter horse × Paso Peruano crosses, with pedigree analysis suggesting that the variant was of quarter horse ancestry [19]. Subsequently, W13 was identified in a family of Miniature Horses in Australia (*n* = 4, 1 stallion and three descendants) [25]. Notably, the phenotype of the sire depicted a white spotting pattern that was not a completely white coat, which contrasts with the phenotype and photographic records of the *W13/N* ponies reported here. In our study, 14 *W13/N* ponies with available photographs had a similar all-white coat with pink skin phenotype as reported by Haase et al., regardless of the other white spotting variants present (Figure 1), demonstrating that W13 results in a Mendelian inherited dominant white phenotype [19]. Therefore, it is possible that the Australian Miniature stallion may have gain of function genetic variant(s) that enables melanocyte survival. This remains to be evaluated.

While the W13 allele frequency identified for the two breeds investigated in this study is low, the presence of W13, its confirmed association with an all-white coat phenotype, and the likelihood that this variant is homozygous lethal support the use of W13 testing for marker-assisted selection. This is especially important when other known variants do not explain coat color phenotype and when breeding two all-white Miniature Horses and/or Shetland ponies to minimize chances of embryonic lethality. Further, given that this report reflects the largest number of animals identified with the W13 variant to date, these data provide evidence that the variant may be found in other not yet evaluated breeds. Therefore, screening for W13 in other breeds and investigating potential origins is warranted.

## Figures and Tables

**Table 1:** Numbers and cause of all-white coat patterning in Miniature Horses and Shetland ponies.

	Total	W13/N	White Explained by Routinely Tested Variants	White with Unknown Cause
All-White Unregistered Shetland Ponies	19	14	4	1 ^a^
Owner-Reported White Miniature Horses	25	2	20	3 ^b^
Owner-Reported White Shetland Ponies	5	0	5	0

^a^ SW1/N, Figure 1e, ^b^ 1 horse was E/E a/a Z/N SW1/N, 1 E/e A/a Cr/Cr O/N, and 1 horse was e/e A/a Cr/N Z/N.

**Table 2:** Genotypes detected during routine testing contributing to white coat pattern phenotypes in Miniature Horses and Shetland ponies.

Genotype	All-White Unregistered Shetland Ponies	Owner-Reported White Miniature Horses	Owner-Reported White Shetland Ponies
*G/_*		1	2
*G/_* + *LP/LP* + *W20/N*		1	
*G/_* + *LP/N* + *TO/N*		1	
*G/_* + *O/N*		1	
*G/_* + *SB1/N*		1	
*G/_* + *TO/N*		2	
*G/_* + *W20/N*	2		
*G/_* + *LP/N* + *SW1/N* + *W20/N*			1
*SB1/SB1*	1	1	1
*SB1/SB1* + *LP/N*	1		
*SB1/SB1* + *SW1/N*		1	
*SB1/SB1* + *O/N*		3	
*SW1/SW1* + *O/N,* + *SB1/N*		1	
*SW1/SW1* + *TO/N*		2	1
*SW1/N* + *O/N* + *SB1/W20*		1	
*SW1/N* + *O/N* + *SB1/N* + *TO/N*		2	
*O/N* + *SB1/W20*		1	
*LP/LP* + *PATN1/N*		1	
Totals	4	20	5

**Table 3:** Genotypes for white patterning loci in Shetland ponies and Miniature Horses with W13.

Genotype	All-White Unregistered Shetland Ponies	Owner-Reported White Miniature Horses
*W13/N*	5	
*W13/N* + *LP/N* + *PATN1/N*	2	
*W13/N* + *TO/N*	1	1
*W13/N* + *SB1/N*	2	
*W13/N* + *SW1/N*	2	1
*W13/N* + *SW1/N* + *PATN1/N*	1	
*W13/N* + *SW1/N* + *W20/N*	1	
Total	14	2

**Table 4:** Allele frequency of W13 in Miniature Horses and Shetland ponies.

Randomly Selected	Total	*W13/N*	W13 Allele Frequency
Miniature Horses	80	1	0.0063
Shetland Ponies	59	0	0

## Data Availability

These data will be made available upon reasonable request to the corresponding author.
